# Identification of ferroptosis-associated tumor antigens as the potential targets to prevent head and neck squamous cell carcinoma

**DOI:** 10.1016/j.gendis.2024.101212

**Published:** 2024-01-19

**Authors:** Qiming Zhai, Zhiwei Wang, Han Tang, Shanshan Hu, Meihua Chen, Ping Ji

**Affiliations:** a Stomatological Hospital of Chongqing Medical University, Chongqing Key Laboratory of Oral Diseases and Biomedical Sciences, Chongqing Municipal Key Laboratory of Oral Biomedical Engineering of Higher Education, Chongqing 401147, China; b Department of Orthodontics, School of Stomatology, Fourth Military Medical University, Xi'an, Shaanxi 710032, China; c Sichuan Cancer Hospital & Institute, Sichuan Cancer Center, School of Medicine, University of Electronic Science and Technology of China, Radiation Oncology Key Laboratory of Sichuan Province, Chengdu, Sichuan 610041, China

**Keywords:** Ferroptosis, Ferroptosis subtypes, Head and neck squamous cell carcinoma, Immunotherapy, Tumor antigens

## Abstract

Head and neck squamous cell carcinoma (HNSC) represents nearly 90% of all head and neck tumors. The current treatment modality for HNSC patients primarily involves surgical intervention and radiotherapy, but its therapeutic efficacy remains limited. The mRNA vaccine based on tumor antigens seems promising for cancer treatment. Ferroptosis, a novel form of cell death, is linked to tumor progression and cancer immunotherapy. Nevertheless, the effectiveness of ferroptosis-associated tumor antigens in treating HNSC remains uncertain. In this study, we identified three ferroptosis-associated tumor antigens, namely caveolin1 (CAV1), ferritin heavy chain (FTH1), and solute carrier 3A2 (SLC3A2), as being overexpressed and mutated based on data obtained from The Cancer Genome Atlas and Gene Expression Omnibus databases. These antigens were strongly associated with poor prognosis and infiltration of antigen-presenting cells in HNSC. We further identified two ferroptosis subtypes (FS1 and FS2) with distinct molecular, cellular, and clinical properties to identify antigen-sensitive individuals. Our findings indicate that FS1 exhibits an immune “hot” phenotype, whereas FS2 displays an immune “cold” phenotype. Additionally, differential expression of immunogenic cell death modulators and immune checkpoints was observed between these two immune subtypes. Further exploration of the HNSC's immune landscape revealed significant heterogeneity among individual patients. Our findings suggest that CAV1, FTH1, and SLC3A2 are potential targets to prevent HNSC in FS2 patients. Overall, our research reveals the potential of ferroptosis-associated mRNA vaccines for HNSC and identifies an effective patient population for vaccine treatment.

## Introduction

Head and neck squamous cell carcinoma (HNSC) ranks sixth worldwide and accounts for 90% of head and neck cancers. HNSC affects over 550,000 people annually, with approximately 300,000 deaths per year.^1^ HNSC includes laryngeal, oral, nasopharyngeal, and hypopharyngeal cancers.^2^ Conventional treatments for HNSC include radiation therapy, chemotherapy, and anti-cancer drug therapy.^3^ Despite these treatments, HNSC, particularly laryngeal squamous cell carcinoma, has poor prognosis and survival rates. As such, it is imperative to develop productive strategies that can improve the therapeutic outcomes for patients with HNSC.

Notably, specific tumor antigens provide the targets for mRNA vaccine in the tumor immunotherapy field.^4^^,^^5^ The primary objective of mRNA vaccination is to stimulate a robust immune response against cancer cells. This is achieved by administering synthetic mRNA that encodes tumor-specific or tumor-associated antigens through engineered autologous dendritic cells *in vitro* or by encapsulated or non-encapsulated mRNA injections.^6^, ^7^, ^8^ Antigen-presenting cells uptake and deliver mRNA to the cytoplasm, where antigens process it to initiate the major histocompatibility complex presentation cascade.^9^ Multiple trials have demonstrated that tumor antigens administered to cancer patients resulted in the development of enduring immunity with manageable toxicities.^10^, ^11^, ^12^, ^13^ Thus, tumor antigens presented by mRNA vaccines may be a promising cancer treatment, especially when combined with other immunotherapeutic approaches. Despite ongoing efforts, the identification of suitable antigens from a vast pool of mutated candidates remains a significant challenge, resulting in the absence of an effective vaccine. Additionally, the categorization of patient sub-populations that are amenable to vaccination has yet to be established.

Ferroptosis, a novel form of cell death mediated by iron-dependent lipid peroxidation accumulation, has been found as a natural tumor suppressor that contributes to tumor development when inactivated.^14^^,^^15^ Notably, research has demonstrated the involvement of ferroptosis in both T-cell immunity and cancer immunotherapy. The induction of ferroptosis in cancer cells can produce a vaccine-like effect, stimulating anti-tumor immunity and overcoming resistance to immunotherapy. Thus, ferroptosis-based mRNA vaccines may treat refractory tumors.

Our research aims to identify HNSC antigens for providing potential targets to prevent HNSC. Additionally, we aim to classify ferroptosis subtypes based on their unique cellular, molecular, and clinical characteristics, which will aid in identifying appropriate patients sensitive to ferroptosis-associated tumor antigens. Our study may provide a theoretical foundation for developing anti-HNSC mRNA vaccines that are tailored to specific patient populations.

## Methods

### Data extraction and processing

The GDC TCGA HNSC cohort, consisting of 546 individuals, was acquired through HNSC dataset retrieval from the UCSC Xena database (https://xena.ucsc.edu). The data was transformed from Count and FPKM formats to TPM format. The somatic mutation data for HNSC patients was obtained from the “Masked Somatic Mutation” dataset, which was preprocessed using VarScan software and visualized using the Maftools R package. We obtained patient clinical data, including age, TNM stage, survival time, and survival status. Moreover, patients without clinical data were excluded, resulting in 603 patients with survival information and 427 patients with other clinical information. The gene expression data of GSE65858^16^ and patient clinical data, including survival time and status, were obtained from the GEO database, with data samples from Homo sapiens and a microarray platform based on GPL10558. This study normalized 270 tumor samples using R's limma package.^17^ Furthermore, 424 ferroptosis-related genes, comprising driver genes, suppressor genes, and markers, were extracted from FerrDB (www.zhounan.org/ferrdb/).^16^ Immunogenic cell death (ICD) and immune checkpoint (ICP) genes were sourced from prior literature.^18^

### GEPIA analysis and cBioPortal analysis

The raw RNA-Seq data obtained from the TCGA database was recalculated using UCSC Xena to ensure data balance and efficient differential analysis. The Gene Expression Profiling Interactive Analysis (GEPIA, http://gepia2.cancer-pku.cn) was used to integrate differential gene expression.^19^ The identification of differentially expressed genes with |log2FC| values >1 and q values <0.01 was performed using analysis of variance. The Kaplan–Meier method was employed to assess overall survival and progression-free survival, with cut-off values set at the median, and comparison was conducted using the log-rank test.

The cBio Cancer Genomics Portal (http://www.cbioportal.org)^20^ was utilized to integrate RNA-seq raw data obtained from databases like TCGA to compare genetic alterations in HNSC. From this analysis, microsatellite instability data and tumor mutation load data were extracted for TCGA-HNSC patients. Statistical significance was determined by a *P*-value threshold of <0.05.

### Identification of potential tumor antigens and immune cell infiltration

ImmuCellAI estimates 24 immune cell abundances using gene expression datasets like RNA-Seq and microarray data. These immune cells comprise 18 T-cell subtypes and six other immune cells, namely B cells, natural killer cells, monocytes, macrophages, neutrophils, and dendritic cells.^21^ ImmuCellAI was used to examine the relationship between dendritic cells/macrophages/B cells and potential tumor antigens. Immune score was calculated by the ESTIMATE website (https://bioinformatics.mdanderson.org/estimate/). A statistical significance level of *P* < 0.05 was employed.

### Identification of the ferroptosis subtypes and validation

The “ConsensusClusterPlus” R package^22^ of ferroptosis-related genes was clustered following expression profiles and a consistency matrix was constructed to identify the corresponding ferroptosis subtypes and gene modules. The partitioning process was executed using a median algorithm with a “1-Pearson correlation” distance metric, with 1000 replications; each replication involved resampling 80% of the patients in the cohort. Using the consensus matrix and cumulative distribution function, the optimal partitioning was identified for clustering sets from 2 to 9. The immune subtypes were then validated in an independent GSE65858 cohort in the same setting.

### Prognostic evaluation of ferroptosis subtypes

A log-rank test was performed to evaluate the prognostic values of ferroptosis subtypes. ANOVA was used to correlate ferroptosis subtypes with different immune-related molecular and cellular features. A chi-square test was used to screen for the most frequently mutated genes. Single-sample GSEA by GSVA package was used to calculate the immune enrichment score for each sample.^23^

### Gene co-expression network

The “WGCNA” R package was applied to screen the modules for immune-related genes.^24^ Using the pickSoftTreshold function, the ideal soft threshold was 4, followed by the construction of a scale-free network, a topology matrix, hierarchical clustering, and Eigengenes calculation. Module Eigengenes were used to create inter-module correlations and hierarchical clustering was performed. Functional enrichment analysis was performed through the Metascape database (www.metascape.org/), including the Kyoto Encyclopedia of Genes and Genomes (KEGG) and Gene Ontology (GO).^25^, ^26^, ^27^ Adjusted *P*-value <0.05 was considered significantly enriched.

### Ferroptosis landscape construction of tumor microenvironment

The Monocle package's Gaussian distribution-based dimensionality reduction function was used to display ferroptosis subtype distribution in individual patients by graph learning-based dimensionality reduction analysis. The maximum number of components was set to 4, and the discriminative dimensionality reduction method of “DDRtree” was applied. The immune landscape was visualized with color-coded functional map cell traces of immune subtypes.

### Statistical analysis

All statistical analyses were performed using R software (version 4.1.1). We used the Wilcoxon rank sum test to compare the differences between the two groups. The Kruskal–Wallis's test was used to evaluate the differences between more than two groups. The Spearman correlation method was used for correlation analysis. *P*-value <0.05 was considered as the threshold of statistical significance.

## Results

### Identification of potential ferroptosis-associated tumor antigens in HNSC

**Figure 1:**
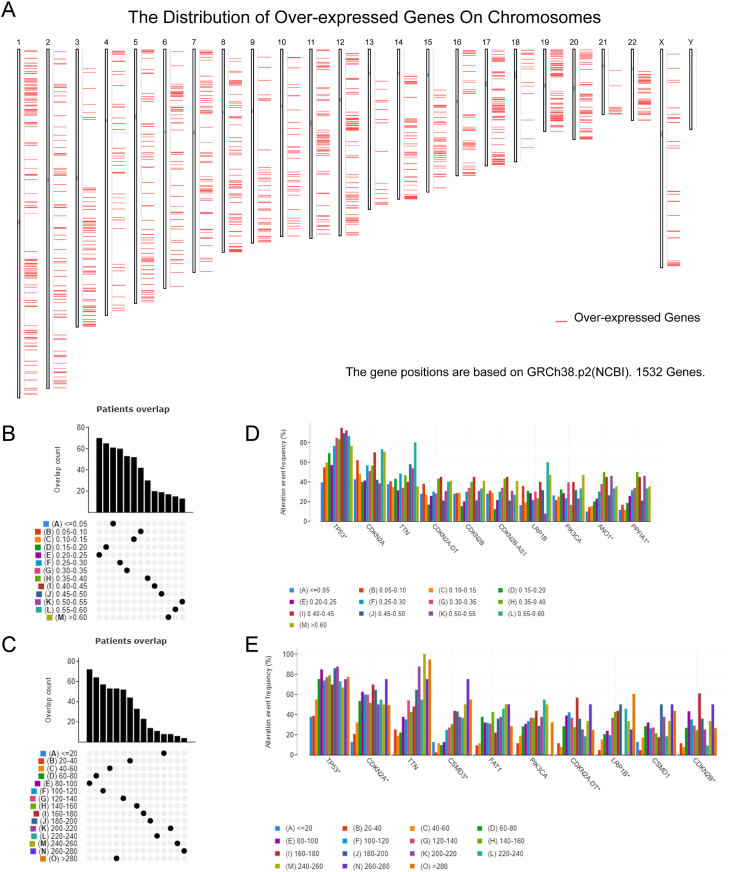
Identification of potential tumor-associated and tumor-specific antigens in head and neck squamous cell carcinoma (HNSC). **(A)** The chromosomal distribution of up- and down-regulated genes in HNSC is suggested. **(B–E)** The samples overlapped in altered genomic segments (B) and mutation count groups (C) and the top ten genes with the highest frequency in altered genomic fragments (D) and mutation count groups (E).

The technical flow chart for the present study is illustrated in Figure S1. To find possible mRNA vaccines for ferroptosis in HNSC, genes with aberrant expression were screened, resulting in 2138 differently expressed genes, 1532 of which could encode tumor-associated antigens for overexpression (Fig. 1A). Subsequently, a comprehensive analysis of mutated gene fragments and mutation counts in individual samples led to the identification of 15,855 mutated genes that potentially encode tumor-specific antigens (Fig. 1B, C). The mutation analysis indicated that TP53 exhibited the highest frequency of mutant genomic fragments and mutation counts, as illustrated in Figure 1D and E. Furthermore, our analysis also found that HNSC had high mutation rates in TP53, CDKN2A, TTN, and CSMD3 in terms of mutation number and frequency. Given its extensive full length, mutations in the TTN gene are unlikely to have a significant impact on HNSC due to its limited specificity. In contrast, mutations in other genes may prove more consequential. Using genetic mutations and gene overexpression, 1160 cancer-related genes were identified as frequently mutated and up-regulated, suggesting they may act as tumor antigens.

**Figure 2:**
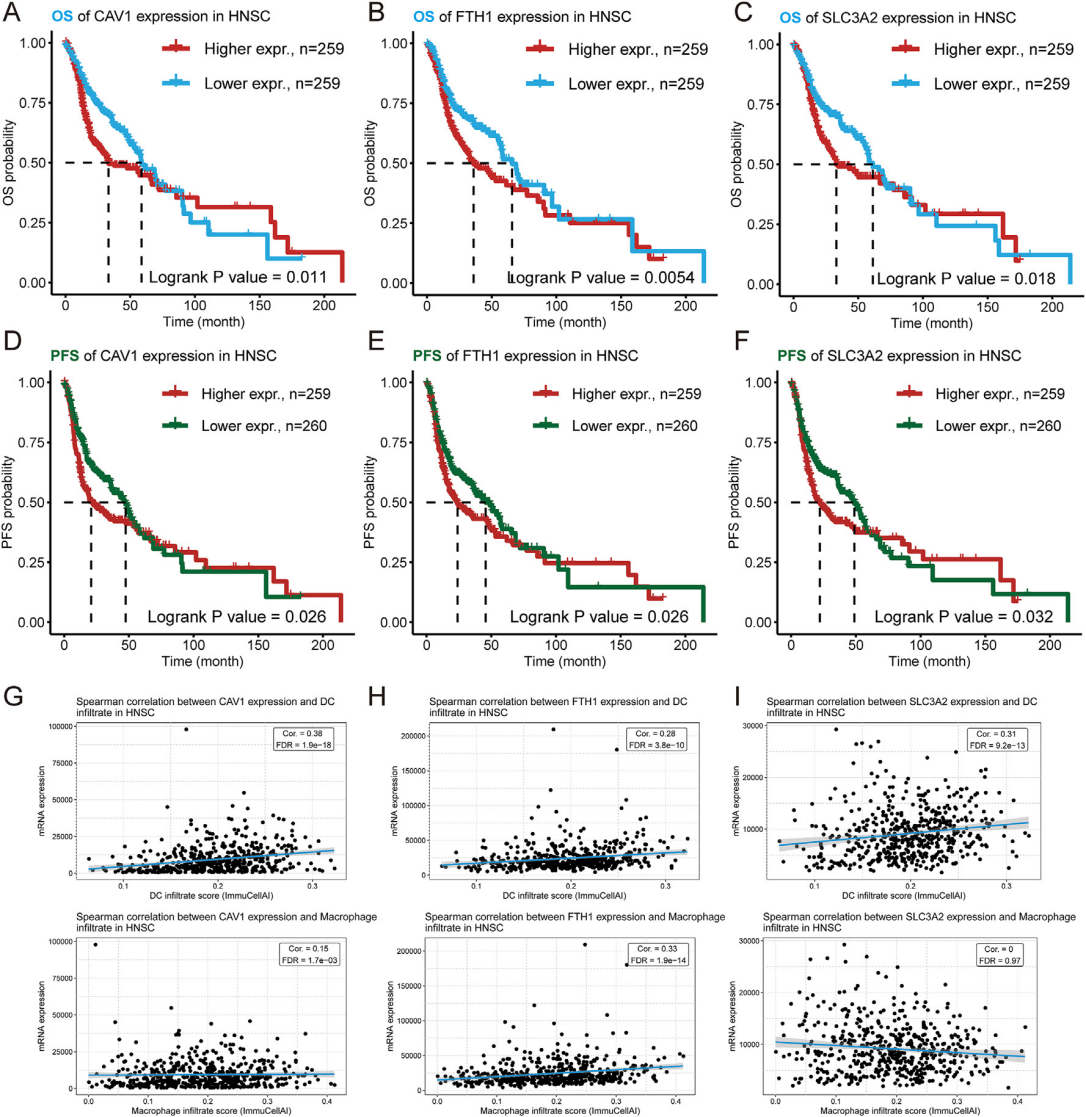
Identification of ferroptosis-relate tumor antigens associated with HNSC prognosis and antigen presentation. **(A–F)** The Kaplan–Meier curves showing overall survival (OS) and progression-free survival (PFS) of head and neck squamous cell carcinoma (HNSC) patients stratified by CAV1 (A, D), FTH1 (B, E), and SLC3A2 (C, F) gene expression levels. (G–I) Correlation analysis of dendritic cells (DCs) and macrophages with CAV1 (G), FTH1 (H), and SLC3A2 (I) by ImmuCellAI calculations.

Subsequently, the focus of this study was directed towards investigating the potential of ferroptosis-related genes as mRNA antigens. Through survival and correlation analysis of antigen-presenting cells, three ferroptosis-related genes, namely CAV1, FTH1, and SLC3A2, were ultimately identified from 1160 potential antigens (Fig. S2). The immunohistochemical analysis showed that HNSC tissues had higher amounts of these three ferroptosis-related proteins than normal tissues (Fig. S3A–C). The findings of the survival analysis indicated that the high expression group of CAV1, FTH1, and SLC3A2 genes had a poorer prognosis for overall survival and progression-free survival (Fig. 2A–F). We also detected the expression of CAV1, FTH1, and SLC3A2 in a normal oral epithelial cell line (NOK) and an oral squamous cell carcinoma epithelial cell line (HN6). As shown in Figure S3D, the ferroptosis-associate antigens were decreased in HN6 compared with NOK, and down-regulated ferroptosis genes were closely related to the faster migration speed of HN6 cells than NOK cells (Fig. S3E). ImmuCellAI demonstrated a positive correlation between CAV1, FTH1, and SLC3A2 genes with dendritic cells and macrophages (Fig. 2G–I) and a negative correlation with B cell expression (Fig. S4A–C). With these observations, it is necessary and effective to give ferroptosis mRNA vaccine therapy to tumor patients to inhibit tumor cell aggressiveness; as a result, the identified ferroptosis-associated antigens may be recognized by antigen-presenting cells and presented to T cells, thereby triggering an immune response. Consequently, these three ferroptosis-related genes may have potential implications in the development of immunotherapeutic strategies.

### Identification of HNSC potential ferroptosis subtypes

**Figure 3:**
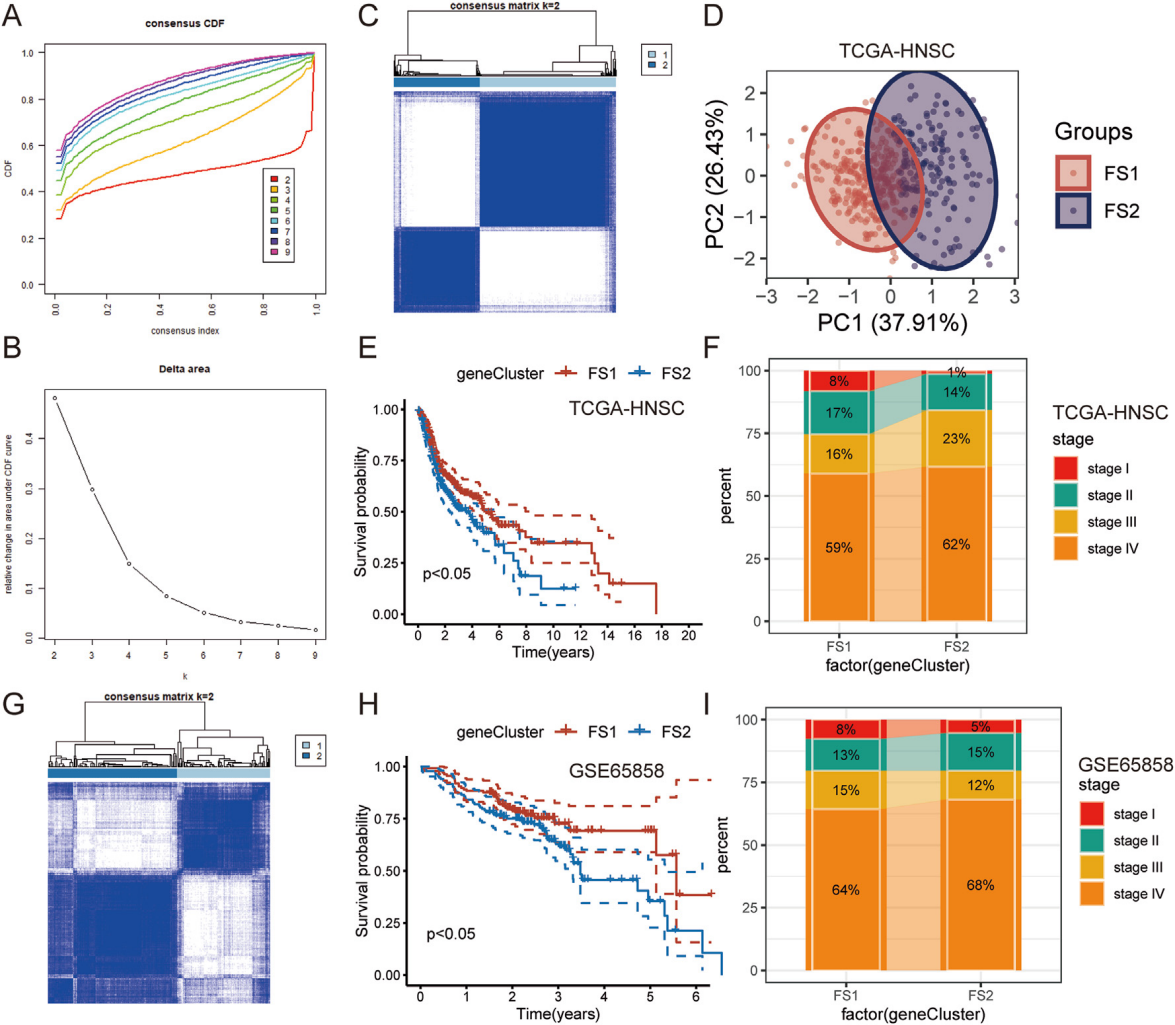
Identification of potential ferroptosis subtypes of head and neck squamous cell carcinoma (HNSC). (A, B) Cumulative distribution function curve (A) and δ area (B) of ferroptosis-related genes in the TCGA-HNSC cohort. (C) Heat map of K = 2 clustering of TCGA-HNSC samples. (D) Two-dimensional principal component analysis (PCA) plot of TCGA-HNSC sample distribution. (E) The Kaplan–Meier curve showing overall survival (OS) of HNSC ferroptosis subtypes in the TCGA-HNSC cohort. (F) The distribution ratio of different staging patients in FS1 and FS2 subtypes based on TCGA-HNSC cohort. (G) Heat map of K = 2 clustering of GSE65868 samples. (H) The Kaplan–Meier curve showing OS of HNSC immune subtypes in the GSE65858 cohort. (I) The distribution ratio of different staging patients in FS1 and FS2 subtypes based on the GSE65757 cohort. HNSC, head and neck squamous cell carcinoma.

Ferroptosis has been shown to impact tumor eradication significantly and has been closely associated with tumor immunity.^28^ Ferroptosis subtypes may indicate the tumor and its microenvironment's immunological state, thereby facilitating the identification of vaccine-eligible patients. Consequently, we analyzed the expression profiles of 424 genes related to ferroptosis in TCGA-HNSC to establish consensus clusters. Utilizing cumulative distribution functions and functional triangle areas, a selection of K = 2 was made, resulting in the stable clustering of ferroptosis-related genes into two subtypes, FS1 and FS2 (Fig. 3A–C). These subtypes showed high diversity in principal component analysis, suggesting their potential utility in classification (Fig. 3D). Furthermore, the Kaplan–Meier survival analysis revealed that the FS2 subtype exhibited a comparatively inferior prognosis compared with the FS1 subtype (Fig. 3E). Furthermore, the distribution of the two ferroptosis subtypes exhibited irregularity across various stages, wherein the FS1 subtype was predominantly observed in early-stage patients. In contrast, the FS2 subtype was more prevalent in mid-to-late-stage patients (Fig. 3F). TCGA-HNSC cohort findings were consistent with those of GSE65858, which also demonstrated the existence of two ferroptosis subtypes (Fig. 3G). The ferroptosis subtype was also prognostically significant in the GSE65858 cohort, with a favorable prognosis observed in FS1 and an unfavorable prognosis in FS2 (Fig. 3H). When comparing various classifications, it was found that a greater percentage of early-stage patients exhibited the FS1 subtype, while a larger proportion of mid-to-late-stage patients displayed the FS2 subtype. Ferroptosis-phenotyping has been shown to predict HNSC prognosis in numerous cohorts.

### Association of ferroptosis subtypes with mutational status

**Figure 4:**
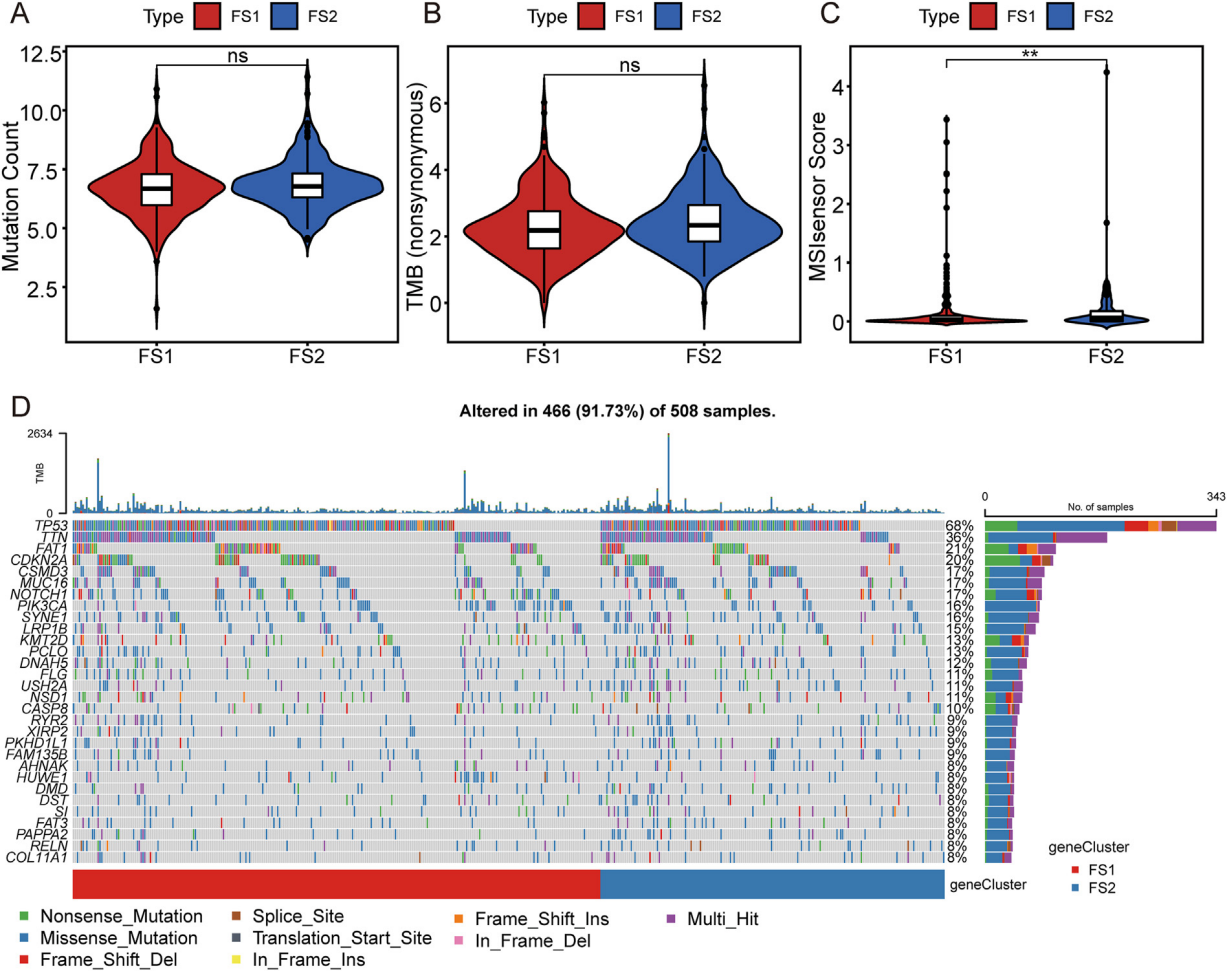
Association of ferroptosis subtypes with tumor mutation burden (TMB) and mutations. **(A–C)** Comparison of mutation count (A), TMB (B), and microsatellite instability (MSI) (C) in TCGA-HNSC in different ferroptosis subtypes. (D) The HNSC waterfall plot showing mutation signature genes in different ferroptosis subtypes. ^∗^*P* < 0.05, ^∗∗^*P* < 0.01, ^∗∗∗^*P* < 0.001.

It has been shown that a heightened tumor mutation burden and somatic mutation rates are linked to improved anti-cancer immunity.^29^ The TCGA-HNSC mutation dataset was used to calculate the tumor mutation burden, microsatellite instability (MSI), and total number of mutations for each HNSC patient and compare two ferroptosis subtypes. The statistical analysis presented in Figure 4 revealed no significant difference in the total number of mutations and mutational load between the two subtypes (Fig. 4A, B). However, the MSI analysis indicated a higher MSI in FS2 compared with FS1 (Fig. 4C). Furthermore, 30 genes, including TP53, TTN, FAT1, and CDKN2A, exhibited varying mutation status in different subtypes (Fig. 4D). The above results suggested that MSI might serve as a potential indicator for the application of mRNA vaccines.

### Relationship between ferroptosis subtypes of HNSC and immunomodulators

**Figure 5:**
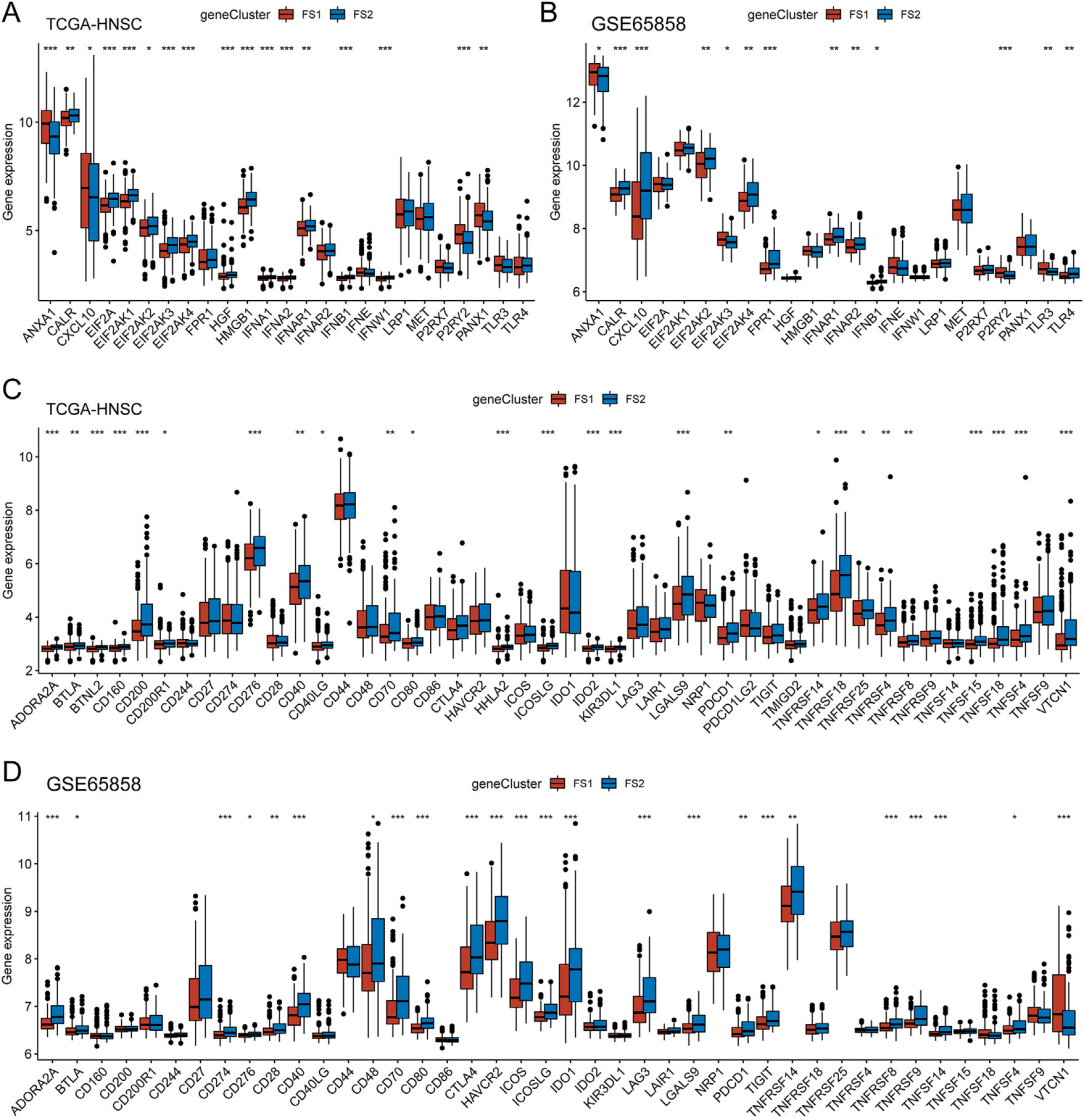
Association of ferroptosis subtypes with ICD and ICP regulatory genes. **(A, B)** Differences in ICD regulator expression between FS1 and FS2 immune subtypes in TCGA-HNSC cohort (A) versus GSE65858 cohort (B). **(C, D)** Differences in ICP expression between IS1 and IS2 immune subtypes in TCGA-HNSC cohort (C) versus GSE65858 cohort (D). ^∗^*P* < 0.05, ^∗∗^*P* < 0.01, ^∗∗∗^*P* < 0.001, ^∗∗∗∗^*P* < 0.0001. ICD, immunogenic cell death; ICP, immune checkpoint.

Our study examined the expression patterns of ICP like PD-L1 and TIM-3 and ICD modulators like CALR and HMGB1 in two ferroptosis subtypes to determine their effects on host anti-tumor immunity and mRNA vaccine efficacy. Analysis of the TCGA-HNSC cohort revealed the presence of 25 ICD genes, of which 17 exhibited differential expression (Fig. 5A). Similarly, the GSE65858 cohort exhibited 23 ICD genes, with 13 demonstrating differential expression (Fig. 5B). Consistent trends in expression were observed for several ICD genes, including ANXA1, CALR, and others, across multiple datasets (Fig. 5A, B). Additionally, a total of 46 ICP genes were identified in the TCGA-HNSC cohort, and 42 ICP genes were examined in the GSE65858 cohort. Notably, most ICPs in both databases expressed similarly, with the FS2 subtype expressing higher than the FS1 subtype (Fig. 5C, D). Thus, ferroptosis-phenotyping may serve as a potential biomarker for mRNA vaccines by reflecting the levels of ICD regulator and ICP expression.

### Cellular and molecular characterization of ferroptosis subtypes

**Figure 6:**
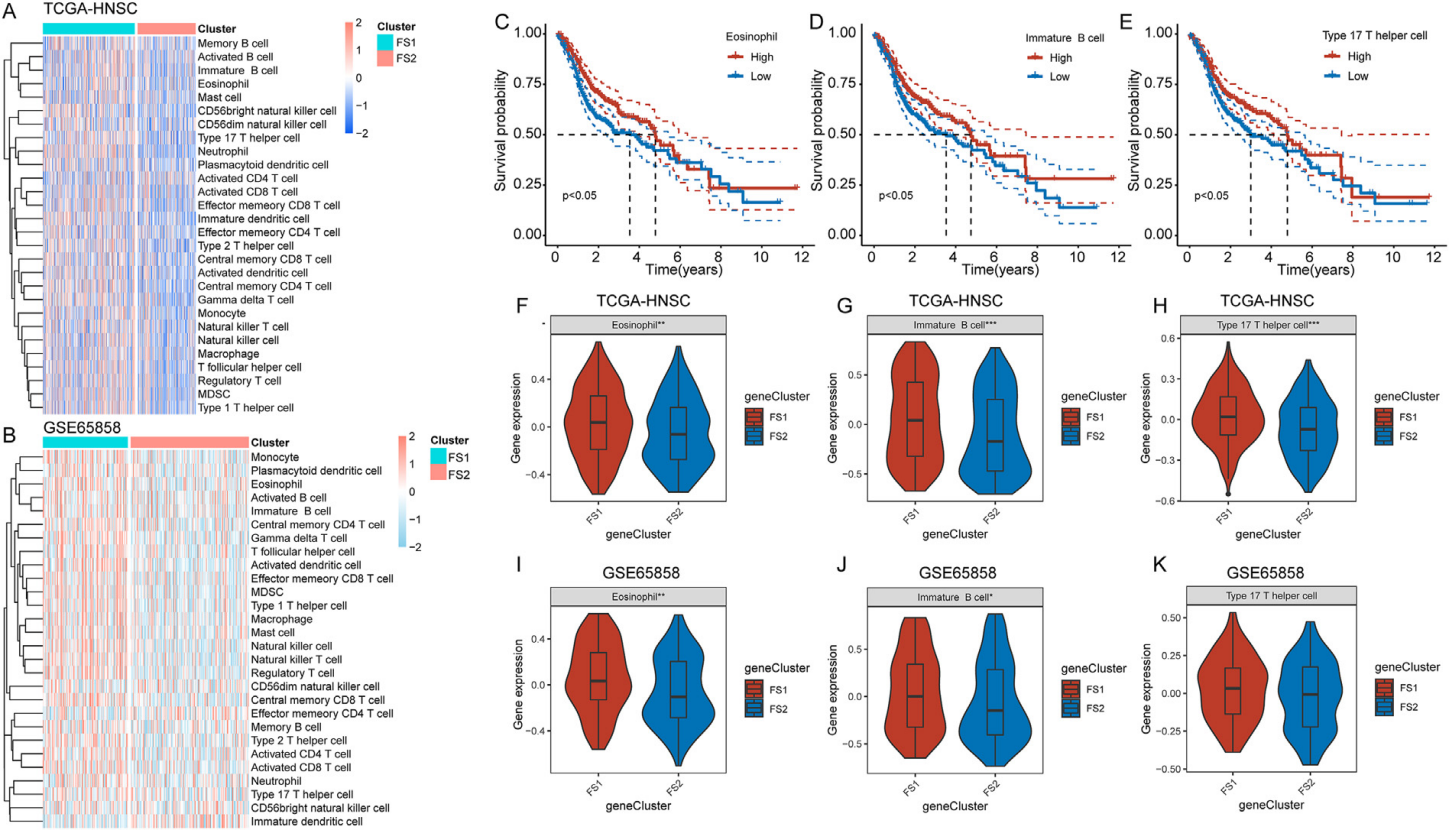
Cellular and molecular characterization of ferroptosis subtypes. **(A, B)** Heat map of enrichment scores of 28 immune cell markers for HNSC ferroptosis subtypes in the TCGA-HNSC (A) and GSE65858 cohorts (B). **(C–E)** The high and low scores of eosinophils (C), immature B cells (D), and type 17 T helper cells (E) in TCGA-PAAD had significant prognostic differences. **(F–H)** Differential enrichment scores of eosinophils (F), immature B cells (G) and type 17 T helper cells (H) in FS1 and FS2 subtypes in TCGA-HNSC. **(I–K)** Differential enrichment scores of eosinophils (I), immature B cells (J), and type 17 T helper cells (K) in GSE65858. ^∗^*P* < 0.05, ^∗∗^*P* < 0.01, ^∗∗∗^*P* < 0.001. HNSC, head and neck squamous cell carcinoma.

The mRNA vaccine's efficacy is contingent upon the tumor's immune state. To elucidate the immune cell constituents of the three immunological subtypes, we employed single-sample GSEA to evaluate 28 signature genes that were previously reported in the TCGA-HNSC and GSE65858 cohorts. Our analysis revealed two distinct clusters of immune cell fractions, as depicted in Figure 6A and B, with discernible discrepancies in immune cell composition across the various subtypes. Among 22 immune cell types, including eosinophils, immature B cells, and type 17 T helper cells, high cell scores indicated a better prognosis (Fig. 6C–E). Both the TCGA-HNSC and GSE65858 datasets showed differences in these three immune cell types' expressions (Fig. 6F–K). Upon applying the ESTIMATE algorithm to calculate immune scores, it was determined that FS2 patients exhibited lower immune cell infiltration compared with FS1 subtypes. At the same time, no statistically significant differences were observed in tumor purity and stromal scores (Fig. S5A–C). The immune trends observed in the GSE65858 dataset were consistent with those observed in TCGA-HNSC. Additionally, higher tumor purity and stromal cell scores were observed in FS1 subtypes (Fig. S5D–F). Consequently, it can be inferred that FS1 subtypes exhibit a “hot” immunological phenotype, while FS2 subtypes exhibit a “cold” immune phenotype. These results provide insight into the immunological state of HNSC immune subgroups and may aid clinicians in identifying patients who would benefit from mRNA vaccination.

### The ferroptosis landscape of HNSC

**Figure 7:**
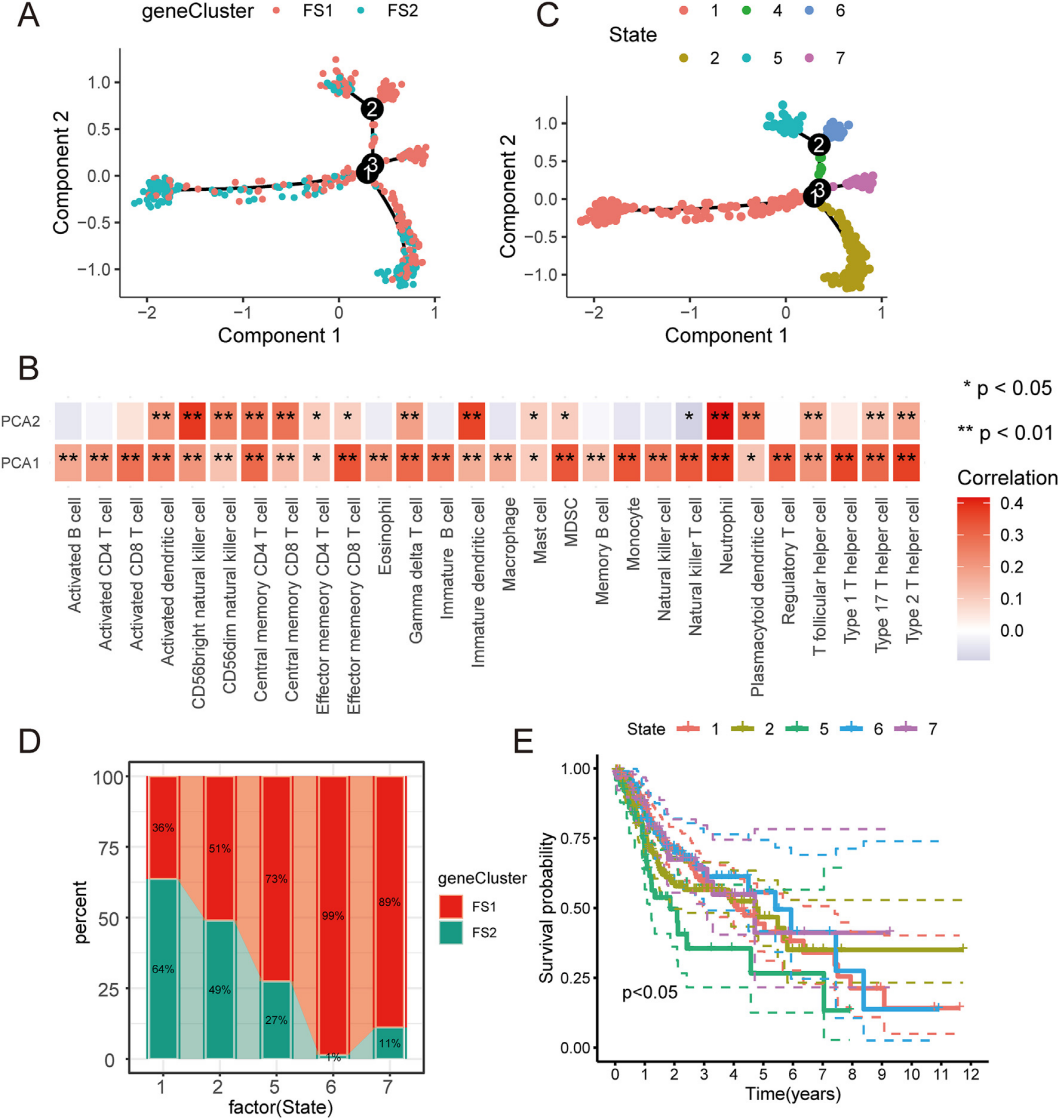
Ferroptosis landscape in head and neck squamous cell carcinoma. **(A)** The position of each patient in the ferroptosis landscape. **(B)** Correlation of PCA1/2 and immunity modules. **(C)** Subpopulation trajectory distribution of the ferroptosis subpopulation of head and neck squamous cell carcinoma. **(D)** The proportion of classified subtypes in different states. **(E)** Prognostic curves of patients with different states. ^∗^*P* < 0.05, ^∗∗^*P* < 0.01, ^∗∗∗^*P* < 0.001. PCA, principal component analysis.

The ferroptosis landscape of HNSC was generated by leveraging the immune gene expression profile of each patient (Fig. 7A). Figure 7B illustrates that principal component analysis 1 on the horizontal axis positively correlated with diverse immune cells, while principal component analysis 2 on the vertical axis positively correlated with most immune cell infiltrations. We also observed considerable intra-cluster variability within the same subtype, leading us to partition the entire sample into seven states based on the location of the sample clusters' trajectories (Fig. 7C). Our analysis focused on states 1, 2, 5, 6, and 7 situated at the endpoints. The proportions of these states in the various ferroptosis subtypes are depicted in Figure 7D. A prognostic analysis revealed a significant difference in overall survival among the survival curves of these five states (Fig. 7E). In summary, immune profiling based on ferroptosis subtypes can effectively determine the early immune status of each HNSC patient and forecast their prognosis, thereby facilitating personalized treatment selection of mRNA vaccines.

### Identification of immune central genes and immune gene co-expression modules in HNSC

**Figure 8:**
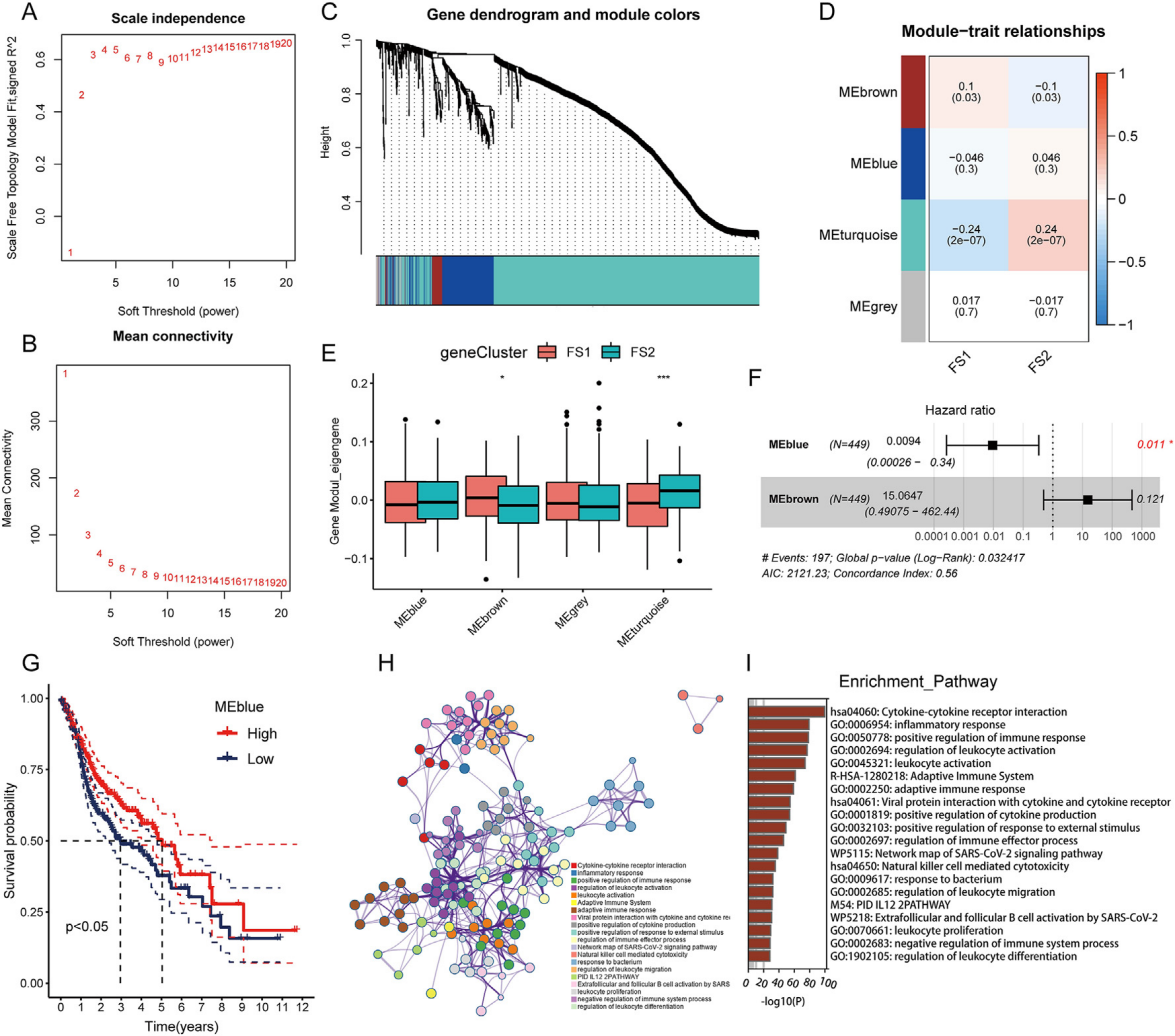
Identification of core immune genes of head and neck squamous cell carcinoma. **(A, B)** The optimal soft threshold was determined by WGCNA. **(C)** Dendrogram of all differentially expressed genes based on dissimilarity measure (1-TOM) clustering. **(D)** The four modules obtained by WGCNA. **(E)** Comparison of immune subtype scores in different modules. **(F)** Multifactor Cox analysis of different module scores. **(G)** Prognostic curves of MEblue. **(H)** The network diagram showing the relationship between the enrichment pathways. **(I)** The bar chart showing the top 20 enriched functions and pathways of genes, including Kyoto Encyclopedia of Genes and Genomes, Gene Ontology, WikiPathways, and Reactome database enrichment analysis results.

Oncologists can assess mRNA vaccine suitability by finding major immune-related genes. We conducted a weighted gene correlation network analysis (WGCNA) of immune-related genes and set the soft threshold at 4 in the scale-free network to find such genes (Fig. 8A, B). We constructed the adjacency matrix and topology matrix of adjacency matrices using the gene matrix. Each gene module was defined by at least 10 genes, and the unique genes within each module were computed and subsequently combined with related modules. Four modules were ultimately obtained, with the gray modules denoting unassigned genes (Fig. 8C, D). Notably, significant disparities were observed between the FS1 and FS2 subtypes regarding eigengene scores across the modules. FS2 had lower scores than FS1 in each module, suggesting an association between FS2 and immunologically cold states (Fig. 8E). Furthermore, an additional prognostic correlation analysis revealed a significant correlation between the blue module and HNSC prognosis (Fig. 8F). Based on the prognosis of blue module scores, the group with high scores demonstrated a significantly superior prognosis compared with the group with low scores (as illustrated in Fig. 8G). As shown in Figure 8H and I, the blue module's genes were enriched in immune-related pathways and functions such as cytokine–cytokine receptor interaction, inflammatory response, positive immune response control, and leukocyte activation regulation. Notably, IL32, FCGR2B, and CD1C were identified within the blue module. Research on hub genes as prognostic markers for HNSC patients and their potential to identify individuals who may benefit from mRNA vaccinations is promising.

## Discussion

HNSC is a highly invasive and metastatic cancer that is prevalent worldwide.^30^^,^^31^ Conventional treatment modalities for HNSC include surgery, anti-cancer drug therapy, and radiation therapy, yet the prognosis for patients remains unfavorable.^31^ Contrarily, mRNA vaccines are currently the subject of extensive basic and clinical investigation. Compared with protein immunization, mRNA vaccination activates a potent stronger CD8^+^ T cell-mediated cellular immunity to boost anti-tumor immunity. Research has demonstrated enhanced therapeutic efficacy when combining tumor vaccines with immune checkpoint inhibitors or chemotherapy agents.^32^ Nevertheless, the development of an effective mRNA vaccine for HNSC remains challenging due to the complexities involved in selecting suitable antigens from a plethora of mutated candidates.

Ferroptosis is a type of regulated cell death involving iron and reactive oxygen species and is unlike other forms of regulated cell death in its unique morphological, biochemical, and genetic features. Recent research has demonstrated that the induction of ferroptosis in cancer cells can stimulate the release of immunostimulatory signals, including damage-associated molecular patterns, which can attract dendritic cells, macrophages, and other immune cells to the site of cell death. This process enhances the cancer cells' immunogenicity and improves the efficacy of immunotherapy for cancer treatment.^33^ To the best of our knowledge, this study represents the initial attempt to screen HNSC antigens utilizing the ferroptosis theory as a basis for the development of an mRNA vaccine. The study involved the construction of aberrantly expressed and mutational landscapes of HNSC, which facilitated the identification of a range of potent antigen targets. Additionally, survival analysis was conducted to ascertain the clinical relevance of the selected antigens, with CAV1, FTH1, and SCL3A2 emerging as promising mRNA candidates associated with favorable prognoses in HNSC. These genes also exhibited substantial positive associations with diverse immune and stromal cells, particularly with macrophages, which directly process and present tumors to T cells, thereby eliciting an immune response. While additional clinical investigations are necessary to assess the effectiveness of these candidates, prior research has substantiated their potential as productive targets for HNSC mRNA vaccines. For instance, CAV1 has been found to be expressed in various solid tumors, including cancerous cells and tumor stroma. It has been associated with cancer progression, metastasis, therapy resistance, and clinical outcomes.^34^ Additionally, CAV1 has been implicated in the induction of acute immune-mediated hepatic damage through the ferroptosis pathway.^35^ The FTH1 expression deregulation, which can be regulated by FTH1 pseudogenes, promoted tumorigenesis.^36^ Furthermore, the SLC3A2 up-regulation in cancer cells has suppressed cystine uptake, leading to an increase in tumor lipid peroxidation, tumor ferroptosis, and tumor regression.^37^

Due to the limited applicability of mRNA vaccines to certain types of HNSC patients, a classification system was developed to categorize patients into two distinct ferroptosis subtypes based on ferroptosis-related genes' expression profiles. This approach aimed to identify patients who would be suitable candidates for mRNA vaccination. Notably, significant prognostic differences were observed between the two ferroptosis subtypes, with FS1 exhibiting superior outcomes in both the TCGA database and GSE65858. Additionally, the two ferroptosis subtypes were unevenly distributed, with FS1 being more common in early-stage patients and FS2 being more common in intermediate- and late-stage patients. These findings suggest that ferroptosis phenotyping may serve as a valuable tool for predicting the prognosis of HNSC patients.

Furthermore, it has been observed that various subtypes of ferroptosis exhibit distinct mutational profiles. Notably, individuals with FS2 tumors exhibit a higher MSI compared with those with FS1 tumors, suggesting that mRNA-based vaccines may be more efficacious in treating FS2 tumors. Conversely, patients with FS1 tumors may demonstrate greater responsiveness to mRNA vaccines when administered with anti-PD1 therapy, particularly considering the high expression of ICP proteins in both the TGCA and GSE65858 cohorts.

The efficacy of mRNA vaccines is contingent upon the immune status of the tumor, which is further elucidated by the immune cell components present in the three immune subtypes. Notably, most immune cell infiltrates exhibited a marked increase in the FS1 subtype, indicating that FS1 is likely an immune “hot” phenotype, whereas FS2 is an immune “cold” phenotype. This finding is supported by the fact that patients with FS2 tumors have a lower overall infiltration of immune and stromal cells and a higher tumor purity than patients with FS1 tumors. Prior research has demonstrated that HNSC tumors impede the immune system through various mechanisms that modulate the function of immune cells, leading to immune evasion and escape.^38^ Therefore, these findings may serve as an indicator of the immune profile of HNSC immune subtypes and identify appropriate candidates for mRNA vaccination. The inclusion of these antigens in an mRNA vaccine may elicit immune infiltration in patients with immunocompetent “cold” IS2 tumors. mRNA vaccines that stimulate the immune system by immune cell infiltration are a promising solution for addressing the problem of insufficient immunogenicity in such tumors.

Notably, as previously noted, it is commonly observed that the “high” mutation status group tends to exhibit an immune “hot” phenotype.^39^^,^^40^ This is attributable to the heightened genomic instability resulting from the increased mutation burden, leading to a greater aberrant manifestation. Consequently, immune cells become more active, thereby manifesting the “hot” phenotype. However, the findings of this study indicate that there is no significant variation in the mutation count and tumor mutation burden. Despite a statistically significant difference in MSI, the magnitude of this difference is minimal, suggesting that it may not enhance immune cell activity and thus characterizing it as a “cold” phenotype. Contrarily, it is important to acknowledge that the correlation between MSI and the F2 subtype in this study may be marginal. Multiple ferroptosis subtypes will be studied to determine how MSI affects tumor phenotypes.

Nevertheless, this study requires further refinement. While our analysis of the mRNA vaccine model is comprehensive, future research should include more meticulous examinations of the treatment effect of the anticipated antigens through the design and synthesis of an mRNA vaccine delivery system. Molecular mechanism research is needed to understand further how the mRNA vaccine works *in vivo*, and we expect clinical implementation improvements.

## Conclusion

This study provides a comprehensive characterization of the ferroptosis mRNA vaccine and identifies a potential target population for vaccine treatment in HNSC. Our findings indicate that CAV1, FTH1, and SLC3A2 are promising targets for the HNSC mRNA vaccine, particularly for patients with FS2 tumors. Our findings provide a theoretical foundation for developing a patient-specific anti-HNSC mRNA vaccine.

## Author contributions

P.J. and M.H.C. conceived the idea of this study. Q.M.Z. performed the bioinformatics analysis and drafted the manuscript. Z.W.W., H.T., and S.S.H. helped with data collection and interpretation. The authors read and approved the final manuscript.

## Conflict of interests

The authors declare no competing interests.

## Funding

This study was sponsored by the 10.13039/501100001809National Natural Science Foundation of China (No. 82201059, 82071115), 10.13039/100018904Beijing Xisike Clinical Oncology Research Foundation (China) (No. Y-XD202001-0024), the Chongqing Postdoctoral Science Special Foundation (China) (No. 2021XM1031), the 10.13039/501100005230Natural Science Foundation of Chongqing, China (No. CSTB2022NSCQ-BHX0003), and 10.13039/501100002858China Postdoctoral Science Foundation (No. 2022M720599).

## Data availability

All data of this article are available from the corresponding authors.
