# Supplementary material for: Identification of ferroptosis-associated tumor antigens as the potential targets to prevent head and neck squamous cell carcinoma
Source: Genes Dis. 2024 Jan 19;11(6):101212. doi: 10.1016/j.gendis.2024.101212 (PMC11403004; doi:10.1016/j.gendis.2024.101212)
Supplement: Multimedia component 1 [file mmc1.docx]

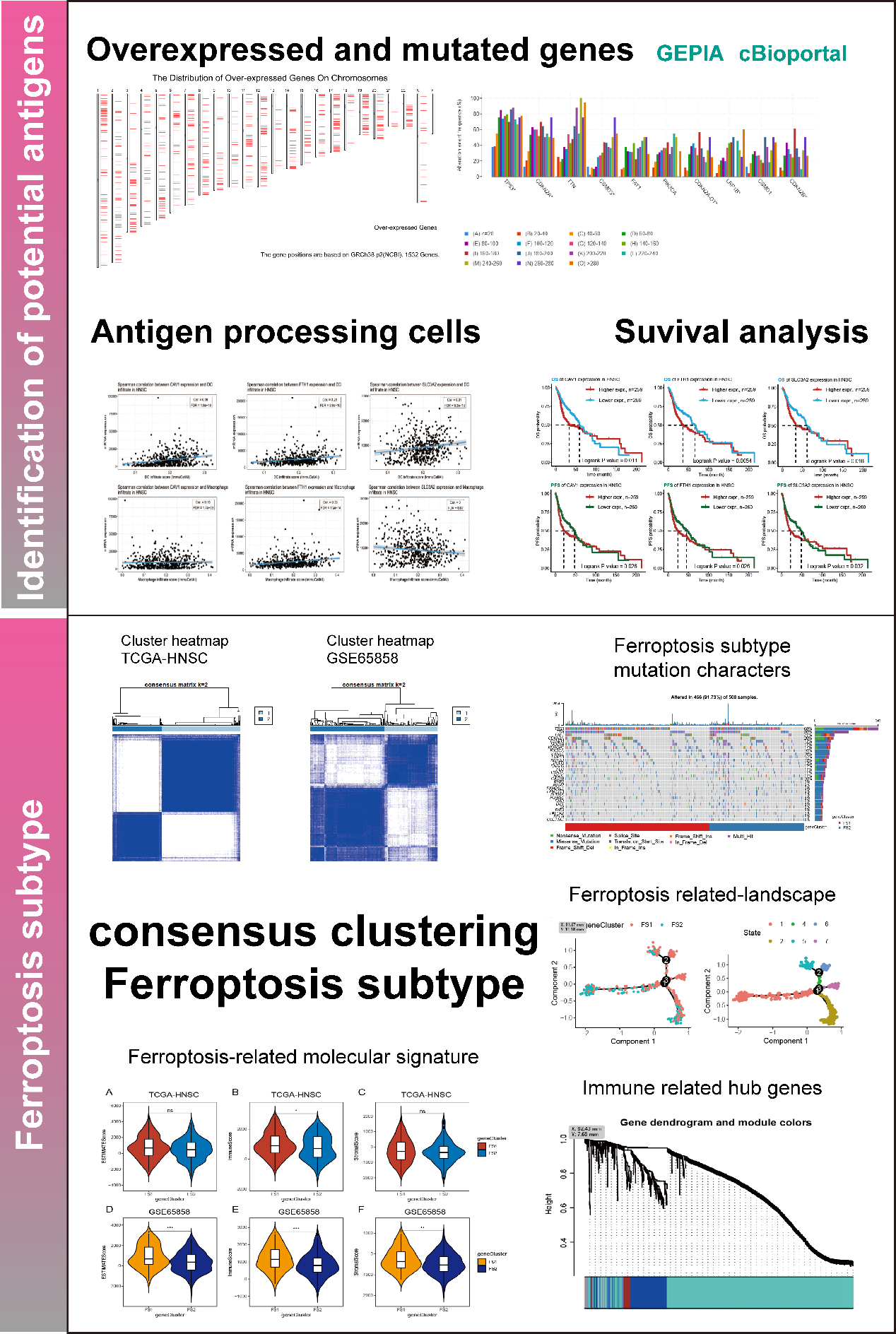


**Fig. S1. The technical flow chart for this study.** Gene expression and clinical information were extracted from the TCGA and GEO databases. ﻿cBioPortal was applied to analyze and visualize genetic alterations. GEPIA was used to calculate the prognostic indices of the selected antigens. TIMER was used to detect the correlation between the infiltration of antigen-presenting cells and the expression of the identified antigens. Consensus clustering analysis was performed for data clustering. Graph learning-based dimensionality reduction analysis was conducted to describe the immune landscape of HNSCC.

**
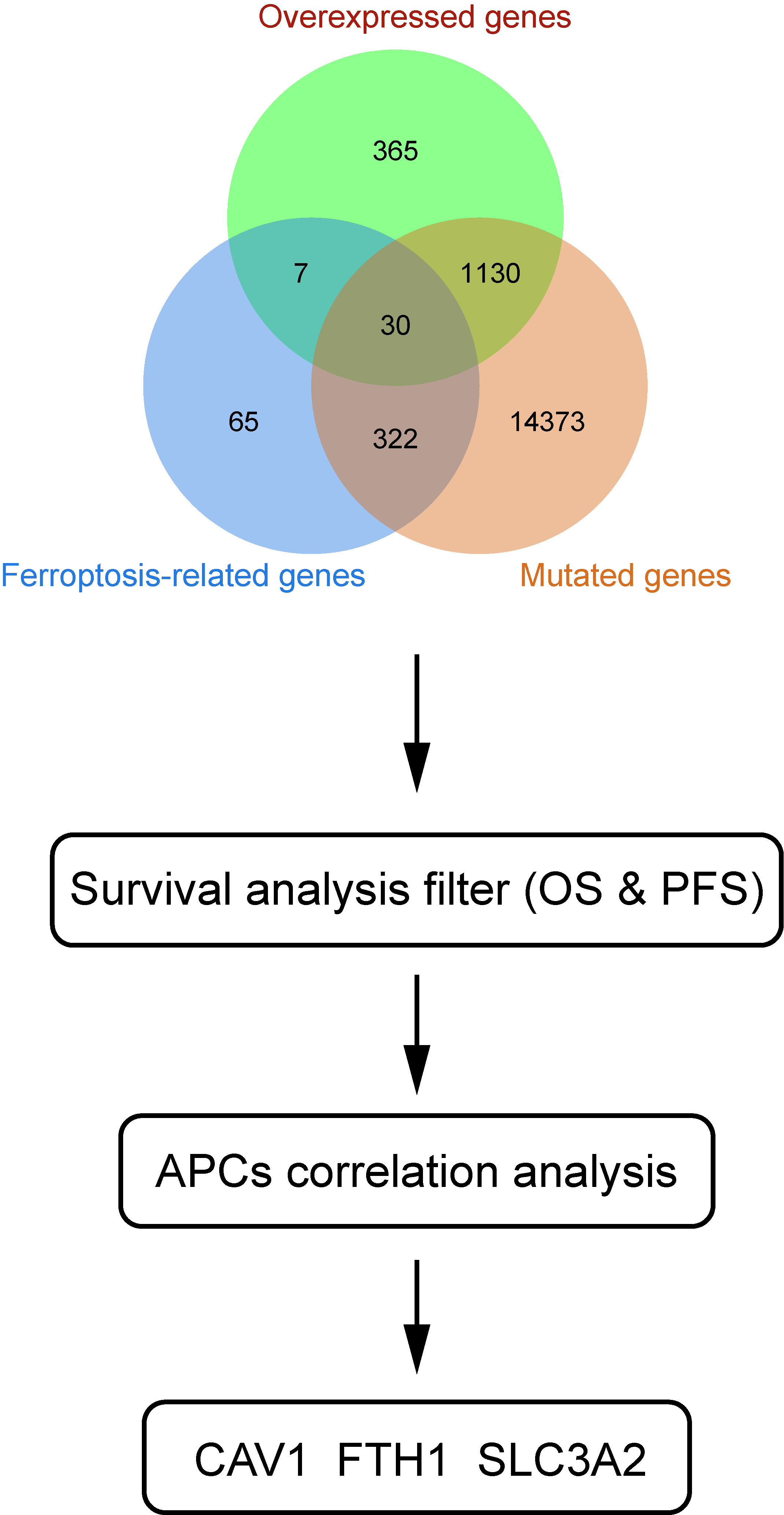
**

**Fig. S2. Ferroptosis-associated antigen screening process in HNSCC.** This flowchart shows how to screen for ferroptosis-associated antigens from the TCGA-HNSCC dataset, including steps like differential, survival, and antigen-presenting cell correlation analysis.

**
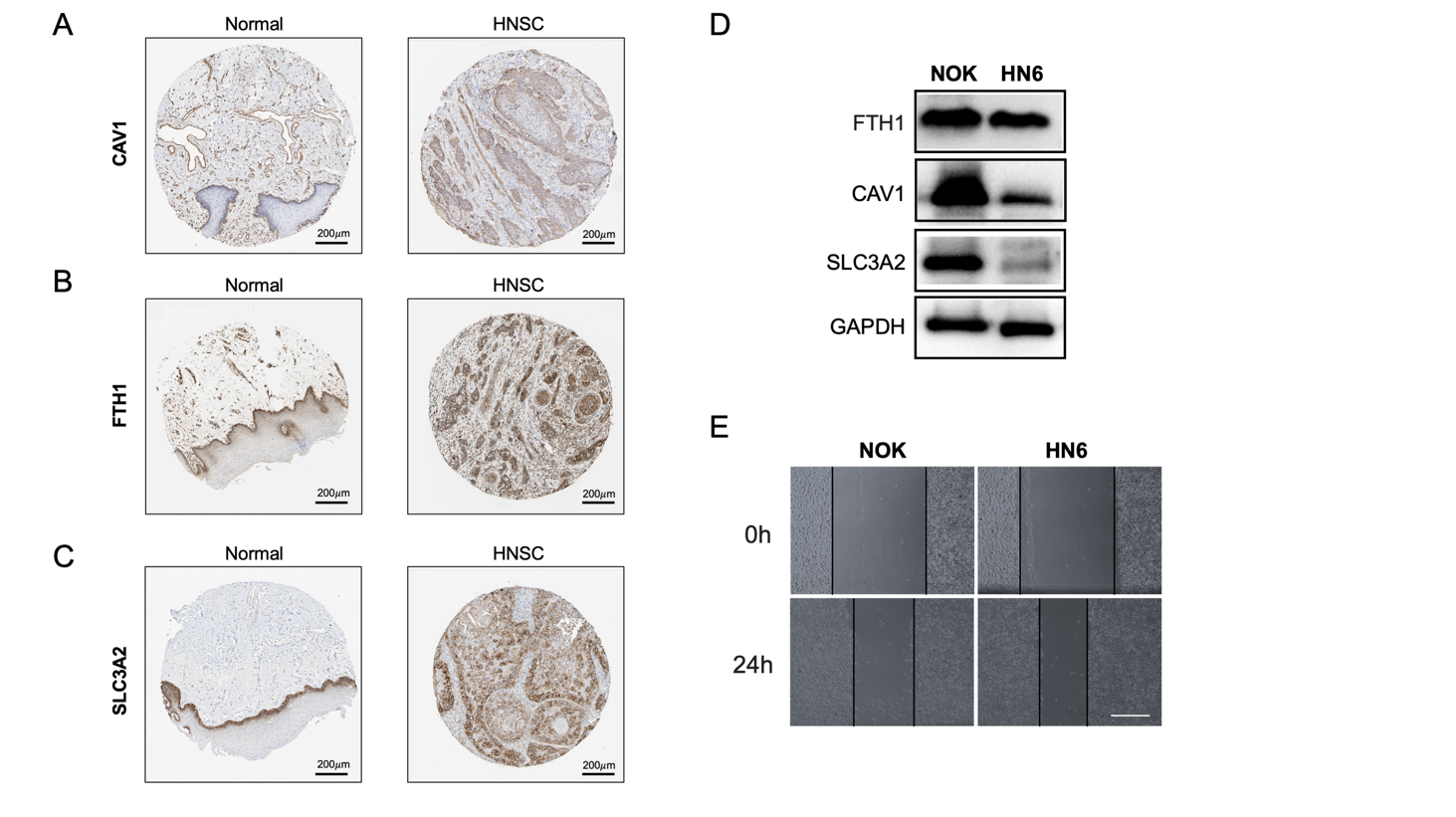
**

**Fig. S3. ﻿Expression of CAV1, FTH1 and SLC3A2 correlates with the phenotype of HNSC. A-C** Expression of CAV1 (A), FTH1 (B), and SLC3A2 (C) was assessed using immunohistochemistry in normal and HNSC tissues. All data are from The Human Protein Altas databases. **D** Western blot analysis of CAV1, FTH1 and SLC3A2 expression in NOK and HN6 cell line. **E** The cell migration ability of NOK and HN6 was determined using wound-healing assays. Scale bars, 400 μm.


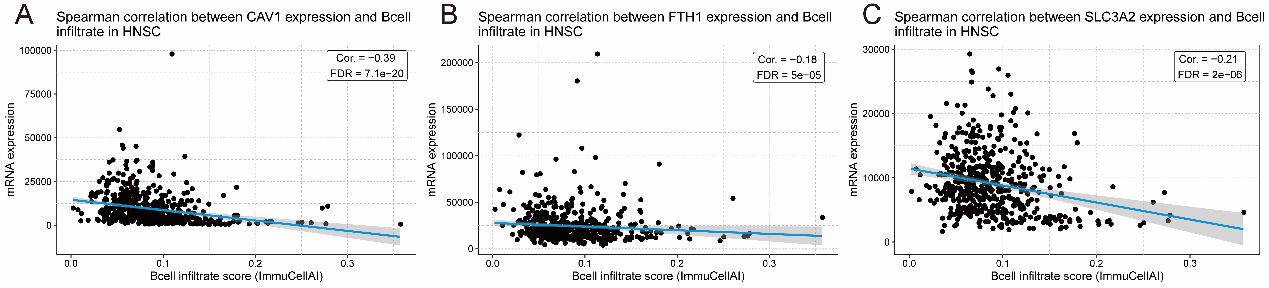


**Fig. S4. Correlation analysis of potential tumor antigens with B cells.** Correlation analysis of B cells with CAV1 (**A**), FTH1 (**B**), and SLC3A2 (**C**) by ImmuCellAI calculations.

**
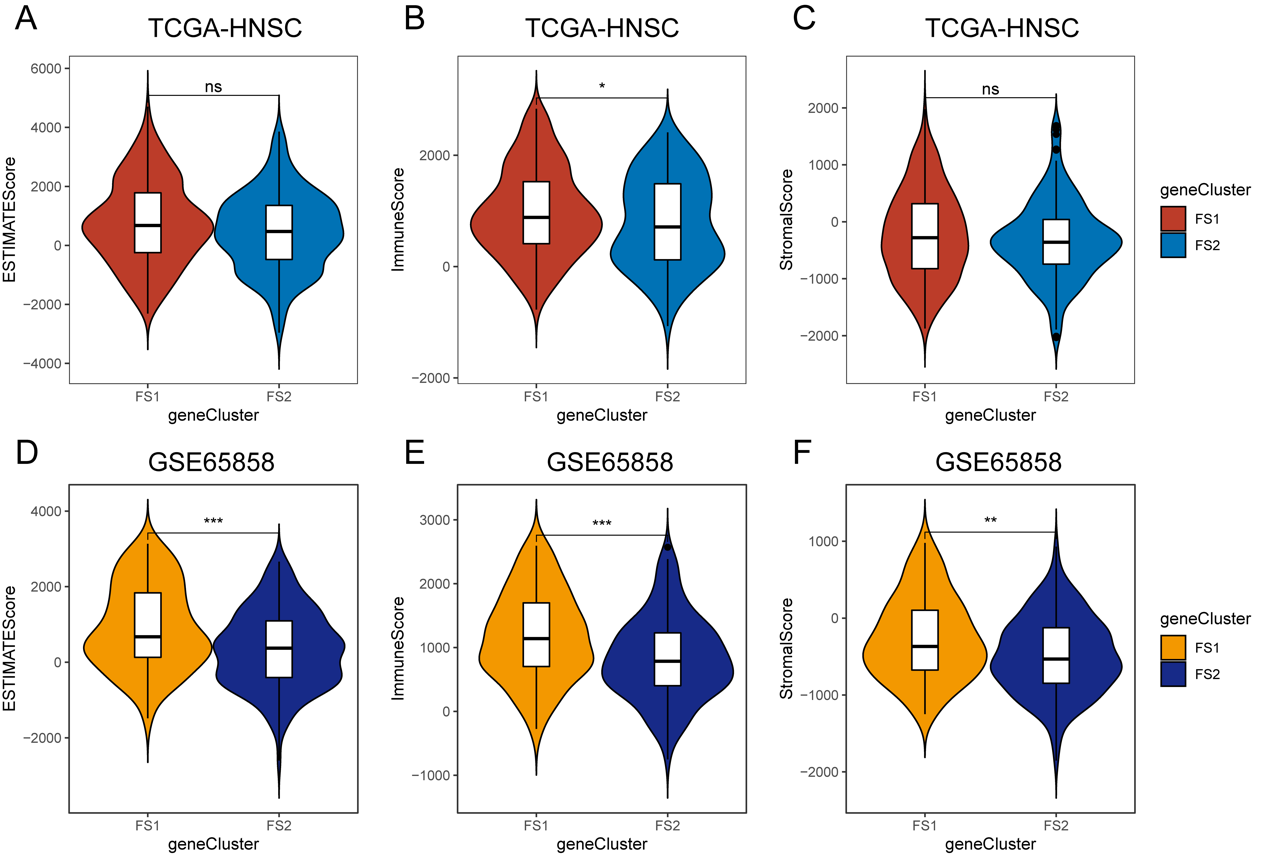
**

**Fig. S5. Comparison of ESTIMATE scores of two ferroptosis subtypes. A-C** Comparison of ESTIAMTE **(A)**, immune score **(B)** and stromal score **(C)** in TCGA-HNSC dataset. **D-F** Comparison of ESTIAMTE **(D)**, immune score **(E)** and stromal score **(F)** in GSE65858 dataset.
